# Mouse glutamate carboxypeptidase II (GCPII) has a similar enzyme activity and inhibition profile but a different tissue distribution to human GCPII

**DOI:** 10.1002/2211-5463.12276

**Published:** 2017-08-29

**Authors:** Tomáš Knedlík, Barbora Vorlová, Václav Navrátil, Jan Tykvart, František Sedlák, Šimon Vaculín, Miloslav Franěk, Pavel Šácha, Jan Konvalinka

**Affiliations:** ^1^ Institute of Organic Chemistry and Biochemistry of the Czech Academy of Sciences Prague Czech Republic; ^2^ Department of Biochemistry Faculty of Science Charles University Prague Czech Republic; ^3^ First Faculty of Medicine Charles University Prague Czech Republic; ^4^ Department of Genetics and Microbiology Faculty of Science Charles University Prague Czech Republic; ^5^ Department of Normal, Pathological and Clinical Physiology Third Faculty of Medicine Charles University Prague Czech Republic; ^6^ Present address: Donnelly Centre for Cellular and Biomolecular Research University of Toronto Toronto ON Canada

**Keywords:** glutamate carboxypeptidase II, mouse animal model, neuronal disorders, prostate cancer, prostate‐specific membrane antigen

## Abstract

Glutamate carboxypeptidase II (GCPII), also known as prostate‐specific membrane antigen (PSMA) or folate hydrolase, is a metallopeptidase expressed predominantly in the human brain and prostate. GCPII expression is considerably increased in prostate carcinoma, and the enzyme also participates in glutamate excitotoxicity in the brain. Therefore, GCPII represents an important diagnostic marker of prostate cancer progression and a putative target for the treatment of both prostate cancer and neuronal disorders associated with glutamate excitotoxicity. For the development of novel therapeutics, mouse models are widely used. However, although mouse GCPII activity has been characterized, a detailed comparison of the enzymatic activity and tissue distribution of the mouse and human GCPII orthologs remains lacking. In this study, we prepared extracellular mouse GCPII and compared it with human GCPII. We found that mouse GCPII possesses lower catalytic efficiency but similar substrate specificity compared with the human protein. Using a panel of GCPII inhibitors, we discovered that inhibition constants are generally similar for mouse and human GCPII. Furthermore, we observed highest expression of GCPII protein in the mouse kidney, brain, and salivary glands. Importantly, we did not detect GCPII in the mouse prostate. Our data suggest that the differences in enzymatic activity and inhibition profile are rather small; therefore, mouse GCPII can approximate human GCPII in drug development and testing. On the other hand, significant differences in GCPII tissue expression must be taken into account when developing novel GCPII‐based anticancer and therapeutic methods, including targeted anticancer drug delivery systems, and when using mice as a model organism.

**Abbreviations**
Avi‐hGCPII: recombinant extracellular human GCPII
Avi‐mGCPII: recombinant extracellular mouse GCPII
GCPII: glutamate carboxypeptidase II
GCPIII: glutamate carboxypeptidase III
NAAG: *N*‐acetyl‐l‐aspartyl‐l‐glutamate
PSMA: prostate‐specific membrane antigen

Glutamate carboxypeptidase II (GCPII; EC3.4.17.21) is a membrane metalloprotease that has been studied intensively over the past 20 years in three different scientific fields: neuroscience, prostate oncology, and dietology. In humans, GCPII is expressed predominantly in the brain 1, 2, prostate 3, 4, small intestine 5, and kidney 4, 6. Because GCPII plays different physiological roles in these tissues, three alternative names for the enzyme have historically been used: *N*‐acetylated alpha‐linked acidic dipeptidase (NAALADase) 7, prostate‐specific membrane antigen (PSMA) 8, and folate hydrolase 5. The close GCPII homolog GCPIII 9, 10, recently identified as β‐citryl‐glutamate hydrolase 11, is also expressed in human tissues.

In the human central nervous system, GCPII hydrolyzes the most abundant peptide neurotransmitter, *N*‐acetyl‐l‐aspartyl‐l‐glutamate (NAAG), into *N*‐acetyl‐l‐aspartate and glutamate 7. Inhibition of this proteolytic activity with selective GCPII inhibitors has been shown to be neuroprotective in experiments with mouse models 12; NAAG activation of metabotropic glutamate type 3 receptors exerts neuroprotective effects toward glutamate‐mediated excitotoxicity caused by elevated levels of glutamate released during stroke, traumatic brain injury, and other pathological conditions 13, 14, 15. In addition to the brain, GCPII is expressed on the human jejunal brush border 5, 16, where it cleaves the terminal glutamates from poly‐γ‐glutamylated folates, enabling their transport across the intestinal mucosa (folate absorption) 17. On the other hand, the function of GCPII in the human prostate is unknown. GCPII is overexpressed in prostate cancer 3, 18; therefore, it has been suggested as a promising target for prostate cancer diagnosis and treatment using targeted strategies 19, 20, 21.

An appropriate animal model is necessary for the development and testing of novel therapeutics. Mice, rats, and pigs are among the most promising candidates to become such a model for GCPII research. Several years ago, our laboratory conducted a study comparing human GCPII with its porcine and rat orthologs 22. The orthologs showed similarity in their enzymatic properties, but considerable differences in terms of their tissue distribution 22. However, mouse GCPII was not included in the study, even though mice now are the most widely used preclinical models for GCPII‐targeted research (stroke 12, traumatic brain injury 23, 24, amyotrophic lateral sclerosis 25, inflammatory, and neuropathic pain 26, 27, reviewed in Refs 13, 28). Therefore, a comparative analysis of mouse GCPII characterization is needed.

Mouse GCPII shares 91% amino acid similarity with human GCPII and preserves the internalization signal MXXXL, despite low similarity in the intracellular domain 29. Mouse GCPII also possesses both NAAG‐hydrolyzing and folate hydrolase activities 29. In contrast to the expression pattern of human GCPII, mouse GCPII is expressed in largest amounts in the kidney and, surprisingly, is absent in the mouse prostate 29. Results from studies with GCPII‐knockout mice have been contradictory: some reports have described normal development to adulthood 9, 30 and others have noted early embryonic death 31, 32.

In the current study, we prepared and characterized recombinant mouse GCPII and compared it with its human counterpart. We put a strong focus on distribution of GCPII in mouse tissues, as this information is highly relevant for the development of novel GCPII‐based anticancer and neuroprotective therapies using mouse models.

## Results

### Efficient one‐step purification method yields purified recombinant mouse GCPII (Avi‐mGCPII)

**Figure 1:**
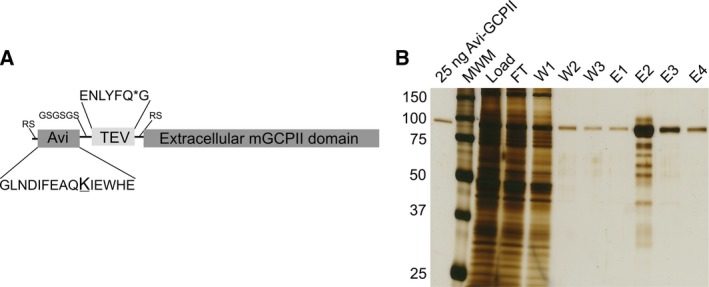
Schematic structure of Avi‐mGCPII and affinity purification. (A) Schematic structure of Avi‐mGCPII containing an Avi sequence (the biotinylated lysine residue is enlarged and underlined) and TEV protease cleavage sequence (the cleavage site is marked with an asterisk). (B) Silver‐stained SDS/PAGE gel showing affinity purification of Avi‐mGCPII expressed in *Drosophila* S2 cells. MWM, molecular weight marker; load, concentrated S2 cell medium; FT, flow‐through; W1–W3, wash fractions; E1–E4, elution fractions. Ten microliter samples was loaded onto the gel, except for the E2 fraction (1 μL was loaded).

As recombinant extracellular human GCPII was shown to correctly represent the endogenous full‐length GCPII 33, 34, we prepared the recombinant extracellular part of mouse GCPII (Avi‐mGCPII) using a *Drosophila* S2 expression system, according to the protocol previously established in our laboratory 34. Avi‐mGCPII has a TEV cleavable Avi‐tag sequence attached to the N terminus of the mouse GCPII extracellular domain (amino acids 45–752), enabling fast one‐step purification (Fig. 1A).

Avi‐mGCPII was purified from the conditioned medium of cells stably transfected with Avi‐mGCPII by affinity chromatography based on the biotin–streptavidin interaction 34, yielding 3 mg of pure protein from 1 L conditioned medium (Fig. 1B).

### Mouse GCPII has lower catalytic efficiency than human GCPII

To characterize the enzyme activity of Avi‐mGCPII, we determined kinetic parameters (*K*_M_ and *k*_cat_) for cleavage of both substrates: *N*‐acetyl‐l‐aspartyl‐l‐glutamate (NAAG) and pteroyl‐di‐l‐glutamate (Table 1). The data revealed that the catalytic efficiency of Avi‐mGCPII is lower than that of its human counterpart. The enzymes had similar turnover numbers but differed in their *K*_M_ values. The differences were more pronounced for pteroyl‐di‐l‐glutamate than for NAAG. Surprisingly, both enzymes had higher catalytic efficiencies for cleavage of pteroyl‐di‐l‐glutamate than for NAAG (Table 1).

**Table 1:** Kinetic parameters of recombinant mouse and human GCPII (Avi‐mGCPII and Avi‐hGCPII, respectively) for their substrates. Kinetic parameters (*K*_M_ and *k*_cat_) of *N*‐acetyl‐l‐aspartyl‐l‐glutamate (NAAG) and pteroyl‐di‐l‐glutamate cleavage were determined using radioenzymatic 34 and HPLC assays 39, respectively. The values shown are mean ± standard deviation of duplicate measurements

Enzymes	NAAG			Pteroyl‐di‐l‐glutamate		
	*K*_M_ [nm]	*k*_cat_ [s^−1^]	*k*_cat_/*K*_M_ [× 10^7 ^s^−1 ^ m]	*K*_M_ [nm]	*k*_cat_ [s^−1^]	*k*_cat_/*K*_M_ [× 10^7 ^s^−1 ^ m]
Avi‐mGCPII	1900 ± 100	1.44 ± 0.02	0.077 ± 0.001	290 ± 20	3.63 ± 0.09	1.26 ± 0.08
Avi‐hGCPII	550 ± 60	1.45 ± 0.04	0.265 ± 0.007	39 ± 2	5.09 ± 0.09	13.2 ± 0.8

Furthermore, to analyze the inhibition profile of Avi‐mGCPII, we determined K_i_ values for several commonly used GCPII inhibitors (using pteroyl‐di‐l‐glutamate as a substrate). The set of GCPII inhibitors included 2‐(phosphonomethyl)pentanedioic acid (2‐PMPA) 35, (S)‐2‐(3‐((S)‐1‐carboxy‐3‐methylbutyl)ureido)pentanedioic acid (ZJ‐43) 36, (*S*)‐2‐(3‐((*S*)‐1‐carboxy‐(4‐iodobenzamido)pentyl)ureido)pentanedioic acid (DCIBzL) 37, quisqualate, DKFZ‐PSMA‐11 38, and beta‐citryl‐l‐glutamate (Table 2). We also tested three compounds recently prepared in our laboratory: JB‐352 and JB‐277 (originally reported as compounds **3** and **22a** 39) and JS‐686 (originally compound **7** 40).

**Table 2:** Inhibition of recombinant mouse and human GCPII (Avi‐mGCPII and Avi‐hGCPII, respectively) by a panel of GCPII inhibitors. Inhibition constants (*K*_*i*_ values) were determined using an HPLC‐based assay using pteroyl‐di‐l‐glutamate as a substrate. The values shown are mean ± standard deviation of duplicate measurements

Compound	*K*_*i*_ (Avi‐mGCPII) (nm)	*K*_*i*_ (Avi‐hGCPII) (nm)
Quisqualate 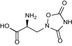	580 ± 60	520 ± 80
2‐PMPA 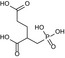	0.56 ± 0.05	0.26 ± 0.03
ZJ‐43 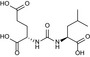	5.9 ± 0.9	0.58 ± 0.07
JB‐352 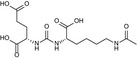	0.66 ± 0.06	0.17 ± 0.04
β‐citryl‐l‐glutamate 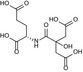	24 000 ± 3000	16 000 ± 5000
DCIBzL 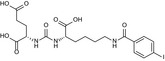	0.028 ± 0.003	0.017 ± 0.002
JB‐277 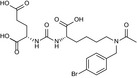	0.68 ± 0.07	0.05 ± 0.02
DKFZ‐PSMA‐11 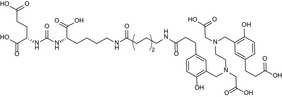	0.10 ± 0.01	0.018 ± 0.002
JS‐686 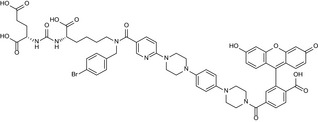	0.049 ± 0.005	0.021 ± 0.004

### Mouse and human GCPII exhibit similar substrate specificities

To obtain information about the substrate specificity of Avi‐mGCPII, we screened 19 different dipeptide libraries of the general formula *N*‐Ac‐A‐X [where A represents a given single N‐terminal amino acid and X represents a mixture of 19 proteinogenic amino acids (all except for cysteine)]. The *N*‐acetylated dipeptide libraries were incubated with the enzymes, and the cleaved amino acids were analyzed by HPLC 41. As a negative control, the potent and selective GCPII inhibitor 2‐PMPA was used to block the specific enzyme activity.

**Figure 2:**
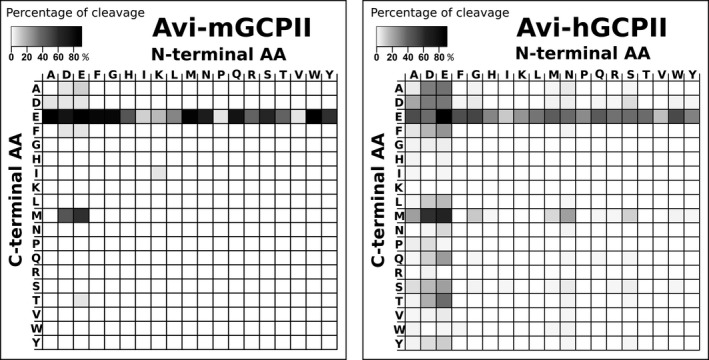
Heat maps representing the substrate specificities of mouse and human GCPII. Recombinant mouse and human GCPII (Avi‐mGCPII and Avi‐hGCPII, respectively) were incubated with 19 dipeptide libraries of the general formula *N*‐Ac‐A‐X‐OH [where A represents a given single N‐terminal amino acid and X represents a mixture of 19 proteinogenic amino acids (all except for cysteine)]. The samples were incubated for 1.5 h at 37 °C, and the cleaved C‐terminal amino acids were quantified using HPLC. As negative controls, experiments either with the GCPII‐specific inhibitor 2‐PMPA or without Avi‐mGCPII/Avi‐hGCPII were performed. The grayscale key represents the percentage of conversion of the particular amino acid in the reaction mixture.

Overall, we found no significant differences in hydrolysis of dipeptide substrates between mouse and human GCPII, as illustrated by heat maps showing mouse and human GCPII processing of individual *N*‐acetylated dipeptides (Fig. 2). The enzymes exhibited a clear preference for glutamate in the C‐terminal position (i.e., glutamate carboxypeptidase activity); mouse GCPII possesses higher selectivity toward the C‐terminal glutamate.

### GCPII is highly expressed in mouse kidney, brain, and major salivary glands

To analyze GCPII distribution in mouse tissues, we collected tissues samples from six mice (three females and three males) and analyzed them by western blot using the anti‐GCPII antibody GCP‐04 2, 42.

**Figure 3:**
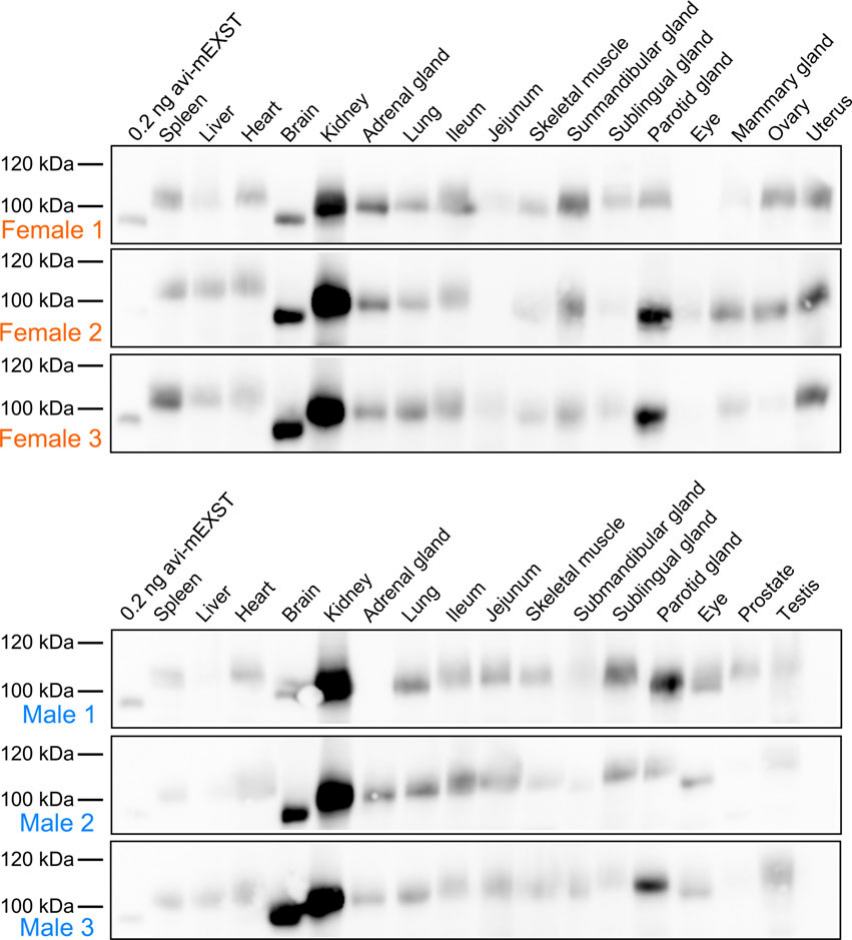
Western blot analysis of GCPII expression in a panel of mouse tissues. Mouse tissue samples (from three males and three females) were homogenized, and lysates were resolved by SDS/PAGE (50 μg of total protein per lane). Mouse GCPII was visualized using the anti‐GCPII primary antibody GCP‐04 2 and HRP‐conjugated goat anti‐mouse secondary antibody.

Mouse GCPII was expressed predominantly in the mouse kidney and brain (Fig. 3), which is in agreement with previous data 9. Interestingly, we observed high and variable expression in the mouse major salivary glands. The different apparent molecular weights are likely caused by different glycosylation of GCPII in the tissues.

**Figure 4:**
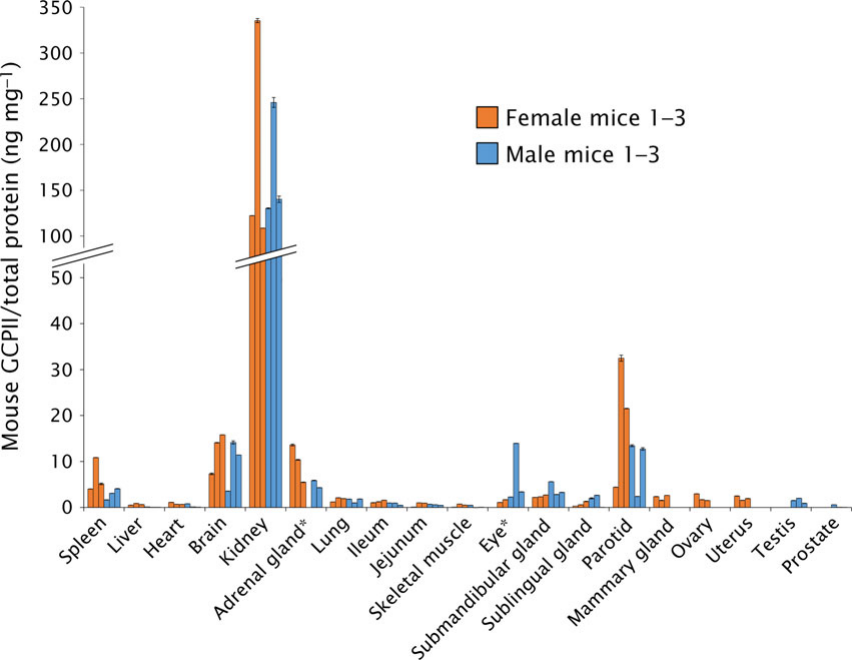
GCPII expression in mouse tissues determined by radioenzymatic assay. The amount of GCPII in mouse tissues was determined by radioenzymatic assay using [^3^H]NAAG as a substrate and recombinant mouse GCPII (Avi‐mGCPII) as a standard. Each tissue sample was measured in duplicate using 1–50 μg total protein in the reaction; the amount of mouse GCPII was normalized to total protein concentration (ng GCPII per mg total protein). The assay was performed with the same tissue samples used in the western blot analysis. *Not determined (adrenal gland: sample M1; eye: sample F1).

We also determined the NAAG‐hydrolyzing activity in the tissue lysate samples using tritium‐labeled NAAG as a substrate and compared these results with the data obtained by western blot analysis. Recombinant mouse GCPII was used as a standard, and the observed levels of NAAG‐hydrolyzing activity were converted to amounts of GCPII, which were then normalized to the total protein concentrations in the homogenates (Fig. 4).

The results confirmed high expression of GCPII in the kidney, brain, and major salivary glands. We did not detect GCPII in the mouse prostate (Fig. 4).

**Figure 5:**
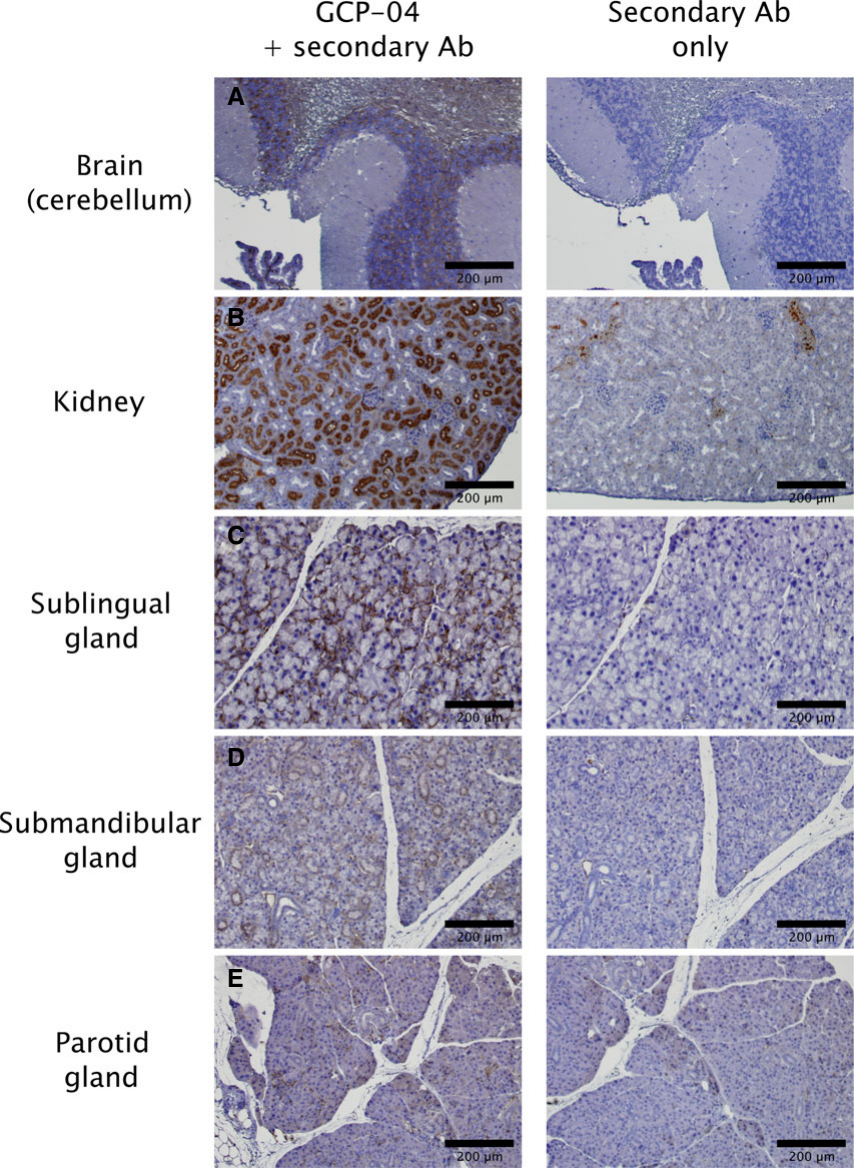
Immunohistochemical staining of chosen mouse tissue sections. Formalin‐fixed, paraffin‐embedded mouse tissue sections were incubated with anti‐GCPII antibody GCP‐04 (at 10 μg·mL^−1^ concentration) to visualize and localize mouse GCPII expression 42. (A) Brain (cerebellum): positive choroid plexus, stratum granulare, white matter. (B) Kidney: positive luminal side of proximal tubules, Bowman capsule; little crossreactivity of secondary anti‐mouse antibody with capillaries and blood vessels could be seen in the negative control. (C) Sublingual gland: positive staining of abluminal cells (probably myoepitelial cells). (D) Submandibular gland: faint staining of intercallated ducts and some non‐glandular abluminal cells. (E) Parotid gland: faint staining of some non‐glandular abluminal cells.

To further examine the location of GCPII in the highly expressing tissues, we performed immunohistochemistry using the anti‐GCPII antibody GCP‐04 2, 42. We found relatively high expression in the white matter in the brain, on luminal side of proximal tubules in the kidney and in the abluminal cells in the major salivary glands (mainly in the sublingual gland) (Fig. 5).

### mRNA expression profile differentiates GCPII and GCPIII expression levels in mouse tissues

**Figure 6:**
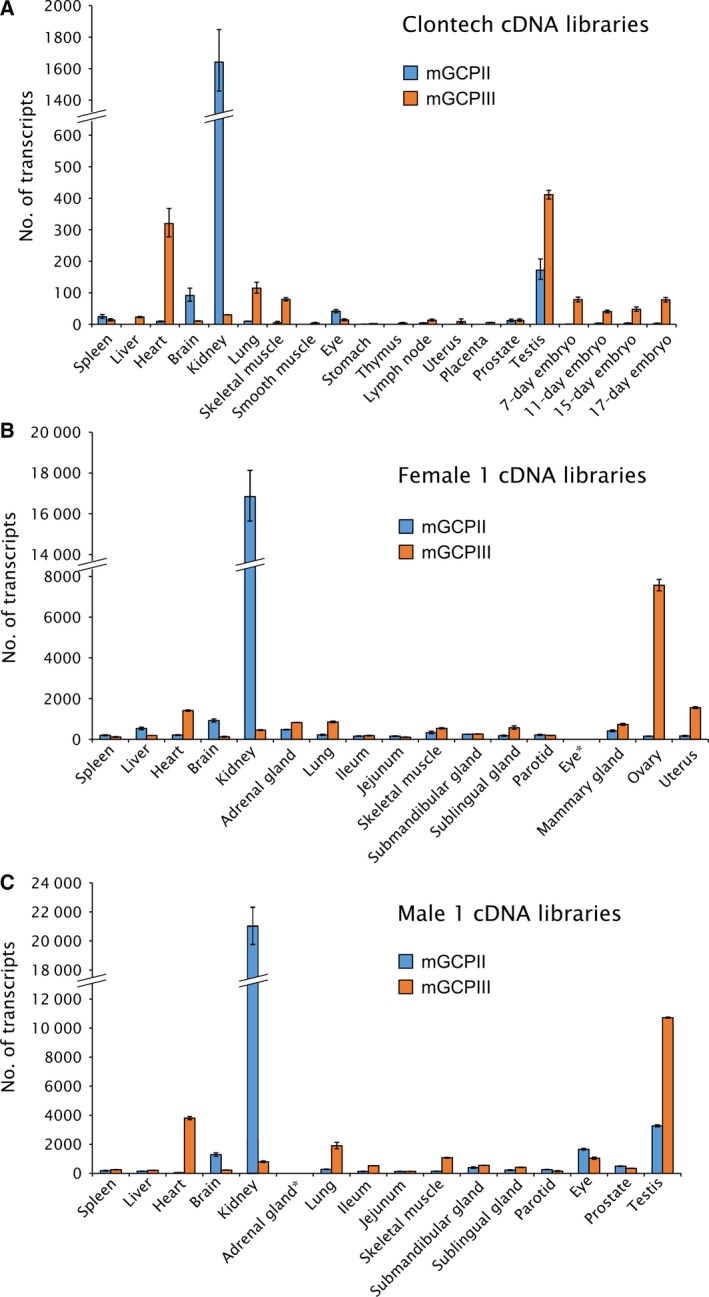
Quantification of mouse GCPII and GCPIII (mGCPII and mGCPIII, respectively) transcripts using qPCR. (A) Quantification of mGCPII and mGCPIII transcripts using qPCR in commercial mouse tissue cDNA libraries from Clontech. The ‘number of transcripts’ corresponds to the amount of transcripts in 1.0 μL of 10‐fold diluted cDNA libraries (for experimental details, see Experimental procedures). Error bars show standard deviations from triplicate measurements. (B, C) Quantification of mGCPII and mGCPIII transcripts using qPCR in cDNA libraries prepared from mouse tissues dissected from one female (B) and one male mouse (C). The ‘number of transcripts’ corresponds to the amount of transcripts per 10 ng of total RNA as a starting material for cDNA synthesis (for experimental details, see Experimental procedures). Error bars show standard deviations from triplicate measurements. *Not determined.

The GCP‐04 antibody cross‐reacts with GCPIII, which also cleaves NAAG 11, 43. Therefore, we decided to further analyze the tissue distribution of both homologs by quantitative RNA determination (qPCR). For these analyses, we used either commercially available panels of mouse tissue cDNA libraries (Fig. 6A) or cDNA libraries prepared from mouse tissues (female 1 and male 1; Fig. 6B,C).

The results from commercial cDNA libraries represent the average tissue distribution of both transcripts in the mouse population. Each library was pooled from several hundred mice and normalized by the vendor to several different housekeeping genes (beta‐actin, G3PDH, phospholipase A2, and ribosomal protein S29). The highest expression of mouse GCPII mRNA was in the kidney, brain, and testis, while mouse GCPIII mRNA was predominantly expressed in the testis, heart, lung, and skeletal muscle (Fig. 6A).

To gain insight into expression of both transcripts in individual mice, we also quantified mRNA transcripts in cDNA libraries prepared from mouse tissues dissected from one female and one male mouse. The results were normalized to the starting amount of total RNA and are in good agreement with findings from the pooled libraries (Fig. 6B,C).

## Discussion

GCPII is a potential pharmaceutical target for a number of pathological conditions caused by glutamate excitotoxicity in the central nervous system, including stroke and traumatic brain injury. Moreover, GCPII has been intensively studied as a target for diagnosis and treatment of prostate cancer, as it is overexpressed in the malignant prostate. In last two decades, a large number of papers have been published describing novel GCPII inhibitors acting as neuroprotective drugs 28, 44 and GCPII inhibitor‐based tools for imaging and/or treating prostate cancer 19, 20, 45, 46, 47. Most of these compounds and methods were evaluated using mouse models. However, there has been no direct comparison of mouse and human GCPII, which would provide important information to assess the usefulness of such mouse models. Therefore, we set out to perform a systematic and detailed study to compare the enzymatic properties of mouse and human GCPII, as well as tissue distributions on both the mRNA and protein levels.

**Figure 7:**
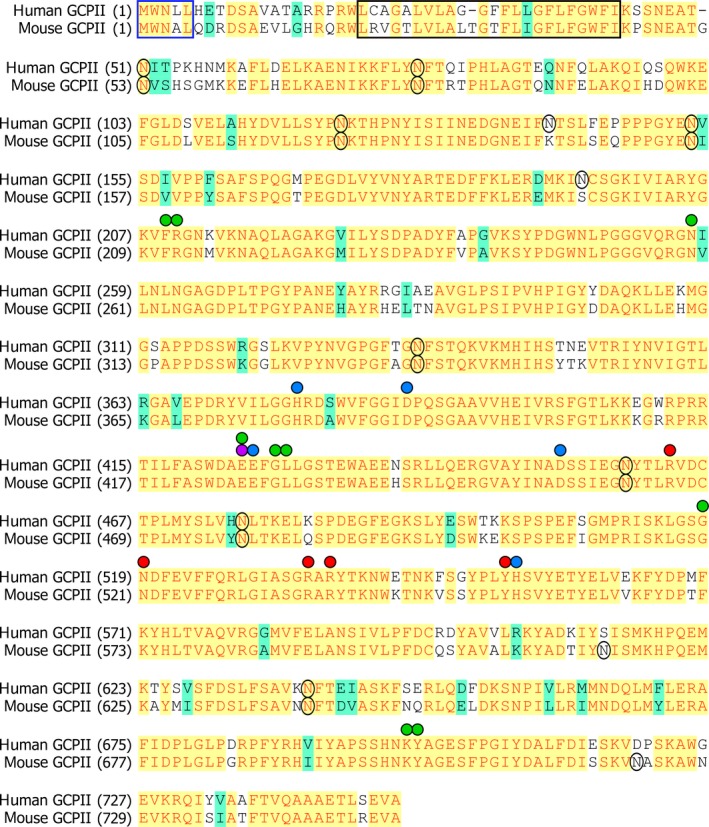
Sequence alignment of the mouse and human GCPII proteins. Identical amino acid residues are highlighted in yellow, similar residues in green, and different residues in white. A blue frame marks the internalization signal MXXXL 54, and a black frame corresponds to predicted GCPII transmembrane domain (predicted by TMHMM Server v. 2.0). Black circles denote potential N‐glycosylation sites (‘N‐X‐S/T’). Green spheres: residues defining the S1′ pocket 49; red spheres: residues forming the S1 pocket 48; purple sphere: proton shuttle catalytic base 55; blue spheres: zinc ligands 56, 57.

We expressed the recombinant extracellular part of mouse GCPII with an N‐terminal Avi‐tag (Avi‐mGCPII), which enables fast and efficient one‐step purification 34. Even though GCPII is a transmembrane enzyme, its extracellular domain is the catalytically active portion and correctly represents endogenous full‐length GCPII 33. To compare the enzymatic properties of mouse and human GCPII, we analyzed the cleavage of their substrates: *N*‐acetyl‐l‐aspartyl‐l‐glutamate (NAAG), which is cleaved by GCPII in the brain, and pteroyl‐di‐l‐glutamate, which is a model substrate for poly‐gamma‐glutamylated folates hydrolyzed by GCPII in the small intestine. Because mouse and human GCPII have high sequence similarity (86% identity and 97% similarity in the extracellular part; Fig. 7), we did not expect to find any significant differences in their enzymatic properties. In fact, we found that while the enzymes are quite similar in NAAG‐hydrolyzing activity, there is an order‐of‐magnitude difference in their catalytic efficiencies for cleavage of pteroyl‐di‐l‐glutamate. This difference lies in the *K*_M_ values of mouse and human GCPII (290 vs. 39 nm for pteroyl‐di‐l‐glutamate and 1900 vs. 550 nm for NAAG). Their turnover numbers are quite similar (3.6 vs. 5.1 s^−1^ and 1.4 vs. 1.5 s^−1^, respectively) (see Table 1). Slightly surprisingly, our data revealed that the catalytic efficiency of NAAG cleavage by mouse and human GCPII is significantly lower than that of pteroyl‐di‐l‐glutamate cleavage (20‐fold and 50‐fold, respectively) (see Table 1).

Additionally, we analyzed the inhibition profile of Avi‐mGCPII using several GCPII inhibitors commonly used in research, including 2‐PMPA, ZJ‐43, DCIBzL, DKFZ‐PSMA‐11, and quisqualate. We also tested several other inhibitors that were prepared in our laboratory. Generally, we did not observe considerable differences in the K_i_ values obtained for mouse and human GCPII. However, inhibitors ZJ‐43 and JB‐277 were exceptions; the K_i_ values for Avi‐mGCPII were 10‐fold higher (Table 2). Both compounds belong to the urea‐based group of GCPII inhibitors, together with DCIBzL. Surprisingly, the K_i_ value of DCIBzL was identical for both enzymes. As seen in Fig. 7, mouse and human GCPII are highly similar and key amino acid residues participating in substrate binding and hydrolysis are identical 48, 49, 50. Thus, in the absence of an experimentally determined structure of mouse GCPII, it is difficult to explain the observed differences in inhibitor binding and catalytic efficiency.

Next, we assessed the substrate specificity of Avi‐mGCPII. We screened dipeptide libraries covering almost all *N*‐acetylated dipeptide substrates, not including cysteine‐containing dipeptides. Unsurprisingly, mouse GCPII exhibited a strong preference for glutamate in the P1′ position, cleaving almost any dipeptide with a C‐terminal glutamate (i.e., glutamate carboxypeptidase activity). It also cleaves dipeptides with methionine in the P1′ position and an acidic amino acid (aspartate or glutamate) in the P1 position. Dipeptides containing any other C‐terminal amino acid were not hydrolyzed by mouse GCPII. The substrate specificity of mouse GCPII thus seems to be even more pronounced than that of the human enzyme (Fig. 2).

In addition to the enzymatic properties of mouse GCPII, its tissue distribution is a relevant aspect both to understand the physiological function of the enzyme and to assess the use of mouse models for targeted drug delivery and GCPII inhibition experiments. Therefore, we set out to elucidate GCPII expression in mouse tissues. To see individual differences, we collected tissue samples from six mice (three females and three males).

To assess GCPII expression on the protein level, we prepared mouse tissue lysates and detected mouse GCPII by western blot using the anti‐GCPII antibody GCP‐04, which was raised against human GCPII 2, 42. Because GCP‐04 recognizes a linear epitope in the GCPII primary structure (amino acids 100–104: WKEFG 22), which is conserved in the mouse GCPII sequence, the antibody can be used for selective and sensitive detection of mouse GCPII as well. We confirmed very high expression of GCPII in the mouse kidney and high expression in the mouse brain, which is in agreement with high GCPII expression in the corresponding human tissues 22. Furthermore, we observed high expression of GCPII in the mouse major salivary glands. Relatively high variability among individual samples of major salivary glands is probably caused by close association of salivary glands that are macroscopically quite similar. This makes the proper dissection of topographically complicated ventral cervical region particularly cumbersome and might lead to cross‐contamination. Therefore, we localized GCPII expression by immunohistochemistry using anti‐GCPII antibody GCP‐04. We observed GCPII expression in all three salivary glands (sublingual, submandibular, and parotid); however, GCPII is expressed predominantly in the sublingual gland, while the expression in the submandibular and parotid glands is lower (Fig. 5).

Mouse prostate contains negligible levels of GCPII, which is consistent with previous findings 9 and in contrast with human prostate, which expresses large amounts of GCPII 6, 22, 51. Additionally, as human and mouse prostates differ considerably in their morphology, we dissected a mouse prostate into its individual parts (anterior, dorsal, and lateral prostate) and searched for potential GCPII expression in each part separately. Nevertheless, we did not detect GCPII expression in any of the tested parts of mouse prostate. Our data suggest that GCPII might also be absent in the mouse jejunum, a tissue where human GCPII cleaves off glutamates from glutamylated folates 17. Rat jejunum and ileum were shown not to contain GCPII, in contrast to the corresponding human tissues, which express large amounts of GCPII 22. Folates in rat intestine are hydrolyzed by γ‐glutamyl hydrolase, not GCPII 52, and the situation in mice may be thus similar.

Furthermore, we verified GCPII tissue distribution obtained by western blot analysis by quantification of NAAG‐hydrolyzing activity in mouse tissues. The activity‐based GCPII expression profile correlated well with the western blot results, confirming strong GCPII expression in the mouse kidney, brain, and salivary glands and no expression in mouse prostate and jejunum.

GCPIII is a close GCPII homolog found both in humans and in mouse. The GCP‐04 antibody also recognizes GCPIII but is roughly 10‐fold less sensitive toward GCPIII than toward GCPII 42. Moreover, GCPIII also hydrolyzes NAAG, although with a lower catalytic efficiency 10, 11, 43. Therefore, GCPII distribution in mouse tissues obtained by western blot analysis with GCP‐04 and activity assay based on NAAG hydrolysis could be distorted by a high amount of GCPIII and low amount of GCPII in a particular tissue. Because there is no specific antibody against GCPIII, we explored GCPII/GCPIII expression in mouse tissues on the mRNA level to differentiate between the two homologs. We quantified GCPII and GCPIII transcripts in both commercially available mouse tissue cDNA libraries and cDNA libraries we prepared from isolated mouse tissues (Fig. 6). Taking these qPCR data into account, GCPIII appears to be the source of the NAAG‐hydrolyzing activity in the mouse ovary, uterus, and heart. GCPIII was most strongly expressed in the testis but was accompanied by rather high quantities of GCPII.

To conclude, we prepared and characterized recombinant mouse GCPII and compared it with human GCPII. We found that the differences in enzymatic activity, inhibition profile, and substrate specificity between mouse and human GCPII are rather small; therefore, mouse GCPII can serve as a suitable substitute for human GCPII in enzymological studies.

Due to the observed lack of GCPII expression in the mouse prostate, mouse might not seem to be an ideal model for the development of prostate cancer diagnostic/therapeutic agents. However, most such studies employ human tumor xenografts in mouse models. For this purpose, mice are generally suitable, because the distribution of GCPII in other tissues is quite similar to that in humans. Therefore, mouse GCPII appears to be a good model for the development of GCPII‐targeted drugs for treatment of prostate cancer and neuronal disorders.

## Experimental procedures

### Cloning of mouse GCPII (Avi‐mGCPII)

The pIRES/mGCPII plasmid encoding full‐length mouse GCPII (amino acids 1‐752) was a kind gift from Warren Heston (Cleveland Clinic, USA).

Because the sequence contained two conflicts compared to the annotated mouse GCPII sequence, we performed site‐directed mutagenesis to remove them (G240A and E287N). The primers 5′‐gctgactactttgttcctGCGgtgaagtcctatcc‐3′ and 5′‐ggataggacttcacCGCaggaacaaagtagtcagc‐3′ were used to remove the sequence conflict at position 240, and the primers 5′‐catgagttgacaAACgctgttggccttc‐3′ and 5′‐gaaggccaacagcGTTtgtcaactcatg‐3′ to remove the sequence conflict at position 287 (changed deoxyribonucleotides are underlined, changed codons capitalized). The mutagenesis was carried out according to the manufacturer's protocol (QuikChange™ Site‐Directed Mutagenesis; Stratagene, San Diego, CA, USA).

Then, the sequence corresponding to the extracellular part of mouse GCPII (amino acids 45‐752) was amplified by PCR using primers 5′‐aaaagatctaaaccttccaatgaagctactgg‐3′ and 5′‐aaactcgagttaagctacttccctcagagtc‐3′ (restriction sites introduced into the sequence are underlined; the primers introduced a *Bgl*II site at the 5′ end and an *Xho*I site at the 3′ end). The resulting DNA fragment was cleaved with *Bgl*II and *Xho*I and ligated into pMT/BiP/AviTEV/rhGCPII plasmid 34 cleaved with the same endonucleases. The correct sequence of the resulting plasmid pMT/BiP/Avi‐mGCPII was verified by DNA sequencing.

### Transfection of *Drosophila* S2 cells and expression of Avi‐mGCPII

*Drosophila* S2 cells expressing BirA biotin‐protein ligase localized in the endoplasmic reticulum (described in Ref. 34) were used to prepare stable Avi‐mGCPII transfectants. The cells were transfected using Calcium Phosphate Transfection Kit (Invitrogen, Waltham, MA, USA) with 9 μg of pMT/BiP/Avi‐mGCPII together with 0.5 μg of pCoBlast (Invitrogen), as previously described 10. The transfected cells were cultivated in the presence of both blasticidin (5 μg·mL^−1^, Invitrogen) and hygromycin B (300 μg·mL^−1^; Invitrogen).

To express Avi‐mGCPII, approximately 2 × 10^6^ stably transfected cells was transferred into a 35‐mm Petri dish supplemented with 2 mL SF900II medium (Invitrogen). The following day, protein expression was induced by adding CuSO_4_ (Sigma‐Aldrich, St. Louis, MO, USA) to a final concentration of 1 mm. After three days, cells were harvested by centrifugation, and the medium was analyzed by western blot.

The large‐scale expression of Avi‐mGCPII was performed as previously described 33. The final volume of cell suspension was 1000 mL.

### Purification of Avi‐mGCPII

Purification of Avi‐mGCPII was performed as previously described 34. Briefly, cell medium (1000 mL) containing secreted biotinylated Avi‐mGCPII was centrifuged at 3400 ***g*** for 45 min. Then, it was concentrated 10‐fold using a LabScale TFF System (Merck Millipore, Billerica, MA, USA) with a Pellicon^®^ XL 50 Cassette, Biomax 100. The concentrated medium was centrifuged again at 3400 ***g*** for 20 min and equilibrated with 300 mm Tris/HCl, 450 mm NaCl, pH 7.2 in a 2 : 1 ratio. The equilibrated concentrated Avi‐mGCPII medium was then mixed with 1 mL Streptavidin Mutein Matrix (Roche, Basel, Switzerland) and incubated with gentle shaking at 6 °C for 15 h. Afterward, the resin was washed with 50 column volumes of 100 mm Tris/HCl, 150 mm NaCl, pH 7.2. Bound biotinylated proteins were eluted with 5 mL of 100 mm Tris/HCl, 150 mm NaCl, 2 mm D‐biotin, pH 7.2, in five consecutive elution fractions (after the first elution fraction, the resin was incubated with elution buffer for 1 h). After regeneration of the resin, the flow‐through fraction was again mixed with the resin, and the purification procedure was repeated.

### Determination of kinetic parameters by radioenzymatic assay

Kinetic parameters (*K*_M_ and *k*_cat_) of *N*‐acetyl‐l‐aspartyl‐l‐glutamate (NAAG) cleavage by Avi‐mGCPII were determined as previously described 34, with a minor modification: The reactions were performed in a 96‐well plate, and appropriate amounts of Avi‐mGCPII were mixed with 25 mm Bis‐Tris propane, 150 mm NaCl, 0.001% octaethylene glycol monododecyl ether (Affymetrix, Santa Clara, CA, USA), pH 7.4.

### Determination of kinetic and inhibition constants by HPLC

Kinetic parameters (*K*_M_ and *k*_cat_) of pteroyl‐di‐l‐glutamate cleavage by Avi‐mGCPII, as well as K_i_ values for all inhibitors, were determined as previously described 39. Briefly, in a 96‐well plate, Avi‐mGCPII was mixed with 25 mm Bis‐Tris propane, 150 mm NaCl, 0.001% octaethylene glycol monododecyl ether (Affymetrix), pH 7.4 (and tested inhibitor, if used), into a final volume of 90 μL. Reactions were started by adding 10 μL of 4 μm pteroyl‐di‐l‐glutamate and incubated at 37 °C for 20 min. The reactions were stopped with 20 μL of 25 μm 2‐PMPA and subsequently analyzed on an Agilent 1200 Series system using an Acquity UPLC HSS T3 1.8 μm column (2.1 × 100 mm; Waters, Milford, MA, USA).

### Animals and tissue isolation

Six C57BL/GJ mice (three males (M) and three females (F)) were sacrificed by cervical dislocation with agreement of the local ethical commission. The ages of the mice were as follows: M1: 5 months; M2: 8 months; M3: 12 months; F1: 8 months; F2 and F3: 12 months. Samples of tissues (for preparation of tissue lysates) were immediately transferred into microtubes and frozen at −80 °C. Samples of tissues (for qPCR quantification) were immediately transferred into RNAlater, impregnated with it for 2 days at 4 °C, and then stored at −80 °C.

### Tissue lysate sample preparation

A small piece of tissue (approx. 30 mg) was transferred into 250 μL of 50 mm Tris/HCl, 100 mm NaCl, pH 7.4, in a 2‐mL microtube. Tissue samples were homogenized using TissueLyser II (30 Hz, 3 min). The homogenates were then diluted with 250 μL of the lysis buffer. Octaethylene glycol monododecyl ether (Affymetrix) was added to reach 1% final concentration, and the homogenate was sonicated in a water bath for 5 min at 0 °C. Finally, the samples were centrifuged at 600 ***g*** for 15 min, and the resulting supernatant was stored at −80 °C until further use. The lysate protein concentration was determined using Bradford 1 × Dye Reagent (Bio‐Rad, Hercules, CA, USA).

### Radioenzymatic determination of NAAG‐hydrolyzing activity in mouse tissues

The determination of NAAG‐hydrolyzing activity in mouse tissues was performed as previously described 53. A sample of tissue lysate was mixed with 20 mm Tris/HCl, 150 mm NaCl, 0.1% Tween 20, pH 7.4, to a final volume of 90 μL. Reactions were started by adding 10 μL of 1 μm NAAG (containing 50 nm tritium‐labeled NAAG), and incubated at 37 °C for 15 h. The reactions were stopped with 100 μL of ice‐cold 200 mm KH_2_PO_4_, 2 mm 2‐mercaptoethanol, pH 7.4. The released glutamate was separated from the unreacted substrate using ion‐exchange AG1‐X resin (Bio‐Rad). The radioactivity of the sample was quantified by liquid scintillation using the Rotiszint ECO Plus scintillation cocktail (Roth) in a Tri‐Carb Liquid Scintillation Counter (Perkin‐Elmer, Waltham, MA, USA). The samples were measured in duplicate.

### SDS/PAGE and western blotting

Protein samples were resolved by reducing SDS/PAGE. Proteins were electroblotted onto a nitrocellulose membrane (wet blotting: 100 V/1 h). After blotting, the membrane was blocked with 0.55% (w/v) casein solution in PBS (Casein Buffer 20X‐4X Concentrate, SDT, Baesweiler, Germany) at room temperature for 1 h. To visualize GCPII, the blots were probed with the antibody GCP‐04 (described in 2) for 12 h at 4 °C (200 ng·mL^−1^; diluted in 0.55% casein solution), washed three times with PBS containing 0.05% Tween 20 (PBST buffer), and incubated with goat anti‐mouse antibody conjugated with horseradish peroxidase (Thermo Scientific, Waltham, MA, USA; diluted in 0.55% casein solution, 1 : 25 000). The blots were then washed three times with PBST to remove unbound antibodies and developed with SuperSignal West Femto Chemiluminescent Substrate (Thermo Scientific). Chemiluminescence was captured with a ChemiDoc‐It™ 600 Imaging System (UVP, Upland, CA, USA).

### Immunohistochemistry

Immunohistochemistry was performed according to the protocol described previously using anti‐GCPII antibody GCP‐04 2 with minor modifications 42. Briefly, after standard histological processing (fixation, dehydration, embedding into paraffin, cutting, paraffin removal, rehydration), heat antigen retrieval was performed using 10 mm sodium citrate, 0.1% Tween 20, pH 6.0 buffer and heating to 110 °C for 15 min in an autoclave. Afterward, samples were incubated in 1.5% hydrogen peroxide solution for 20 min to reduce endogenous peroxidase activity and in 10% fetal bovine serum in PBS to block unspecific interactions. The slides were then stained by primary anti‐GCPII antibody GCP‐04 (10 μg·mL^−1^ in 4 °C, overnight), followed by extensive washing (five times with PBS containing 0.1% Tween 20) and incubation with secondary antibody Histofine^®^ Simple Stain™ MAX PO (MULTI) (Nichirei Bioscience Inc., Tokyo, Japan) diluted 1 : 2 with 10% fetal bovine serum in PBS at room temperature for 1 h. After further extensive washing (five times with PBS containing 0.1% Tween 20), GCPII was visualized using DAB/Plus kit (Diagnostic BioSystems, Pleasanton, CA, USA, 60 s). The slides were counterstained with Harris’ hematoxylin and mounted in polyvinyl alcohol‐based media.

### Carboxypeptidase activity assay

Carboxypeptidase activity (i.e., substrate specificity) of mouse GCPII was determined using *N*‐Ac‐A‐X peptide libraries according to a previously published method 41. Briefly, 1.2 μg Avi‐mGCPII was diluted into 25 mm Bis‐Tris propane, 150 mm NaCl, 0.001% octaethylene glycol monododecyl ether (Affymetrix), pH 7.4, and incubated in the presence of 25 μm dipeptide for 1.5 h at 37 °C. As negative controls, reactions without the enzyme and in the presence of 1 mm 2‐PMPA, a highly selective GCPII inhibitor, were performed. The reaction mixture was then analyzed using HPLC, as previously described 41.

### Total RNA isolation and reverse transcription

First, tissue samples were transferred from RNA later solution (Invitrogen, #AM7021) to RLT buffer (part of the RNEasy Mini Kit, Qiagen, Hilden, Germany, #74106) supplied with β‐mercaptoethanol and homogenized with 5‐mm steel beads (Qiagen; #69989) using TissueLyser II (#85300; Qiagen). Total RNA was isolated using RNeasy Mini Kit according to the manufacturer's instructions. The concentration and purity of isolated RNA were determined spectrophotometrically using a Nanodrop ND‐1000 spectrophotometer. The integrity of each RNA sample was analyzed using the Agilent RNA 6000 Nano kit run on an Agilent 2100 Bioanalyzer. Only samples without significant degradation were used for subsequent steps.

RNA was then reverse‐transcribed using M‐MLV (#28025013; Invitrogen) according to the manufacturer's instructions. Each 20 μL reaction contained up to 2 μg total RNA, 2.5 μm oligo(dT)_20_ primers (#18418020; Invitrogen), 50 ng random hexamers (100 ng if more than 1 μg RNA was transcribed), 40 units of RNAseOUT, 200 units of M‐MLV reverse transcriptase, and other components as specified by the manufacturer.

### Quantitative PCR (qPCR) analysis

All qPCRs were carried out in triplicate in FrameStar 480/96 multiwell plates (#4ti‐0951; 4titude, Wotton, UK) sealed with adhesive optical foil (#4729692001; Roche) using a LightCycler 480 II instrument (Roche) in a total volume of 10 μL. Each reaction consisted of LightCycler 480 Probe Master (Roche) diluted according to the manufacturer's instructions, forward and reverse primers (1 μm final concentration each), fluorescent probe (see description of individual assays for final concentration), and 1 μL of sample or template DNA (positive and nontemplate controls as well as interplate calibrators were included on each plate). Initial denaturation for 3 min at 95 °C was followed by 45 cycles of 10 s at 95 °C, 30 s at 66 °C, and 30 s at 72 °C. The threshold cycle numbers (*C*_q_) were then determined from fluorescence intensities acquired during the qPCR runs by the second‐derivative maximum method using LightCycler 480 software (Roche). The presence and size of PCR products were analyzed by agarose gel electrophoresis.

The amount of mouse GCPII (encoded by the gene Folh1) was quantified by an assay set of forward and reverse primers (sequences 5′‐gattgccagatatgggaaagtg‐3′ and 5′‐cctgccagttgagcattttt‐3′) and fluorescent hydrolysis probe #6 from the Roche Universal Probe Library (LNA octamer sequence 5′‐cagaggaa‐3′; final concentration 100 nm). This set was designed to amplify nucleotides 714–773 in mouse GCPII transcript NM_016770 to yield an amplified product of 60 bps, which spans the region of exons 5 and 6 and corresponds to amino acids 202–223 in the longest open reading frame (ORF). This assay should not amplify genomic sequence because it spans a 1029‐bp intron.

The amount of mouse GCPIII transcript (encoded by the gene Naalad2) was quantified by an assay set of forward and reverse primers (sequences 5′‐aatgatgcagagagactattacgc‐3′ and 5′‐ccagcttttgtctggtggag‐3′) and fluorescent hydrolysis probe #52 from the Roche Universal Probe Library (LNA octamer sequence 5′‐gggaggag‐3′; final concentration 50 nm). This set was designed to amplify nucleotides 922–981 in mouse GCPIII transcript NM_028279 to yield an amplified product of 60 bps, which spans the region of exons 7 and 8 and corresponds to amino acids 289–309 in the longest ORF. This assay should not amplify genomic sequence because it spans a 880‐bp intron.

As a standard for absolute quantification, serial 10‐fold dilutions covering concentrations from 10^8^ to 10^2^ copies per reaction of either pcDNA4 plasmid with subcloned coding sequence of full‐length mouse GCPII (longest ORF from NM_016770 coding amino acids 1–752) or pMT/BiP plasmid with subcloned coding sequence of extracellular part of mouse GCPIII (part of longest ORF from NM_028279 coding amino acids 36–740) were amplified with the corresponding assay set. The initial concentration of plasmid DNA (purified by QIAprep Spin Miniprep Kit, #27106; Qiagen) prior to dilution was determined spectrophotometrically at 260 nm (Nanodrop ND‐1000; Thermo Scientific).

To enable precise absolute comparison between the determined amounts of both transcripts, obtained calibration curves were further normalized against each other by quantification of a common region of both plasmids. The region containing the ampicillin resistance gene was quantified by a set of primers with sequences 5′‐gcagaagtggtcctgcaact‐3′ and 5′‐agcttcccggcaacaatta‐3′ and fluorescent hydrolysis probe #58 from the Roche Universal Probe Library (final concentration 50 nm). In this way, two calibration curves were obtained for each plasmid, one for the amplification of target transcript and one for the common sequence. Finally, the slope and intercept values of both curves were transformed for each plasmid so that the transformed slope and intercept values of the curves for the common sequence were equal between the two plasmids and corresponded to the average value between the two plasmids.

The amount of both transcripts was determined in the prepared tissue cDNA libraries. In each qPCR, an amount of cDNA corresponding to the starting amount of total RNA of 5–10 ng was used, and the amount of GCPII and GCPIII transcripts were normalized to the total amount of RNA. Both transcripts were also quantified in 1.0 μL of 10‐fold diluted commercial tissue cDNA libraries (Mouse MTC Panels I and III supplied by Clontech, Mountain View, CA, USA, #636745 and 636757), which had been normalized to several control genes by the vendor (beta‐actin, G3PDH, phospholipase A2, and ribosomal protein S29).

### Statistical analysis

All values are presented as the mean ± standard deviation.

## Author contributions

The manuscript was written through contributions of all authors. All authors have given approval to the final version of the manuscript. JK and PS conceived the project and analyzed data, TK, BV, JT, VN, PS and JK wrote the manuscript, TK, BV, VN, JT, FS, SV and MF designed, performed and interpreted the experiments.
